# Anemia in pregnancy: a systematic review and meta-analysis of prevalence, determinants, and health impacts in Egypt

**DOI:** 10.1186/s12884-024-07111-9

**Published:** 2025-01-14

**Authors:** Ahmed Azzam, Heba Khaled, Alrefaey K. Alrefaey, Amar Basil, Sarah Ibrahim, Mohamed S. Elsayed, Muhammad Khattab, Nashwa Nabil, Esraa Abdalwanees, Hala Waheed Abdel Halim

**Affiliations:** 1 Department of Microbiology and Immunology, Faculty of Pharmacy, Helwan University, Cairo, Egypt; 2 Department of Biochemistry, Faculty of Pharmacy, Cairo University, Cairo, Egypt; 3 Department of Anesthesia, Intensive Care, and Pain Management, Faculty of Medicine, Mansoura University, Mansoura, Egypt; 4 Intern, Bashir Hospital, Amman, Jordan; 5 Primary Care Physician, Urban Medical Center, Cairo, Egypt; 6 Pharmacist, Mansoura University Children’s Hospital, Mansoura, Egypt; 7 Specialty Doctor in Surgery, Yeovil District Hospital, NHS Foundation Trust, Somerset, UK; 8 Department of Community, Environmental and Occupational Medicine, Faculty of Medicine, Benha University, Benha, Egypt; 9 Pediatric Specialist, Ain Alkhaleej Hospital, Al Ain City, Abu Dhabi, UAE; 10 Department of Obstetrics and Gynecology, Faculty of Medicine for Girls, Al-Azhar University, Cairo, Egypt

**Keywords:** Anemia, Pregnancy, Determinants, Complications, Apgar, Egypt, Meta-analysis

## Abstract

**Background:**

The WHO considers anemia in pregnancy a severe public health issue when prevalence surpasses 40%. In response, we conducted a systematic review and meta-analysis to examine anemia among pregnant women in Egypt, focusing on its prevalence, determinants, and associated complications.

**Methods:**

We conducted a systematic literature search for studies published between January 1, 2010, and August 18, 2024, to identify studies from Egypt reporting on anemia in pregnant women, including its prevalence, associated determinants, and complications. A meta-analysis was conducted using a random-effects model to estimate pooled prevalence, odds ratios (OR), and standardized mean differences (SMD). Sensitivity analyses and publication bias were performed. All statistical analyses were conducted using R software.

**Results:**

Eighteen studies met the eligibility criteria with a total sample size of 14,548. The overall prevalence of anemia among pregnant women was 49% (95% CI: 42–57), with no significant difference between Upper and Lower Egypt (*P* = 0.66). The sensitivity analysis demonstrated the absence of influential outliers and Egger’s test indicated no evidence of publication bias (*P* = 0.17). Anemia prevalence was significantly higher in the third trimester (65%) compared to the second trimester (47%) (*P* = 0.03). Among anemic pregnant women, most cases were mild (47%) and moderate (47%). The determinants of anemia among pregnant women included being over 30 years old (OR: 1.95), residing in rural areas (OR: 1.76), illiteracy (OR: 1.93), birth spacing < 2 years (OR: 2.04), lack of iron supplementation (OR: 2.59), presence of intestinal parasites (OR: 1.38), antenatal visits < 5 (OR: 5.27), multiparity, and low income, all with statistical significance (*p* < 0.05). Regarding dietary determinants, a low intake of meat, vegetables, fruits, and high tea consumption was consistently associated with a higher risk of anemia. For neonatal complications, infants born to anemic mothers had significantly lower Apgar scores, gestational ages, and birth weights (*P* < 0.05), with birth weight being the most adversely impacted (SMD = -1.3).

**Conclusions:**

This meta-analysis shows 49% anemia prevalence in pregnant Egyptian women, indicating severe health concern. The findings highlight the urgent need for targeted interventions aimed at addressing the key determinants identified in this study.

**Supplementary Information:**

The online version contains supplementary material available at 10.1186/s12884-024-07111-9.

## Introduction

Anemia during pregnancy poses a significant public health challenge, particularly in developing countries [1–3]. The World Health Organization (WHO) categorizes anemia in pregnant women by its public health impact: a prevalence below 5% is considered insignificant, 5–19% signals a mild public health issue, 20–39% reflects a moderate concern, and a prevalence of 40% or more constitutes a severe public health crisis [4]. This classification is crucial, as the clinical consequences of anemia in pregnancy are serious, impacting both the mother and the newborn. For instance, it has been associated with a higher risk of neonatal complications, including preterm birth, low birth weight (LBW), and high small for gestational age (SGA) infants [5–7]. On the maternal side, anemic mothers are more likely to experience conditions such as hypertension, diabetes, placental abruption, or chorioamnionitis, and they face a higher risk of requiring blood transfusions, intensive care unit admission, and an increased likelihood of cesarean delivery [5–7].

The prevalence of anemia among pregnant women varies widely, with the highest rates found in low-income countries [1, 8]. In addition, significant disparities between nations are apparent, further highlighting the global inequality in maternal health outcomes. For instance, previous country-specific meta-analyses estimated the prevalence of anemia among pregnant women to be 31.6% in Ethiopia [9], 53% in Sudan [10], and 15% in Iran [11]. This country-based variation may stem from disparities in healthcare access and infrastructure, socioeconomic status, knowledge, and attitudes toward prenatal care and anemia prevention.

To the best of our knowledge, no previous meta-analysis has specifically assessed the prevalence of anemia among pregnant women in Egypt. To overcome the limitations of individual studies and enhance statistical power, we conducted this systematic review and meta-analysis to provide a comprehensive understanding of the epidemiology of anemia in pregnancy, including its determinants and associated neonatal and maternal complications. This study offers essential evidence to support programmatic efforts aimed at addressing anemia in pregnancy in Egypt and guiding future research and policy development.

## Method

### Search strategy

A comprehensive literature search was conducted for studies published between January 1, 2010, and August 18, 2024, using the following databases: PubMed, Scopus, Google Scholar, Web of Science, and the Egyptian Knowledge Bank. In addition, reference lists of the included studies were reviewed to ensure thorough coverage of the relevant literature. Table S1 outlines the detailed search strategy used for each database in this review, including specific keywords and Boolean operators, with adjustments tailored to each database’s search requirements. A library was created to compile articles retrieved from databases, with duplicates removed using Zotero version 6. The remaining articles were then screened for eligibility based on the inclusion criteria, first by title, then by abstract and full text. Although this study was not registered, a protocol was developed and strictly followed without modifications. This systematic review followed the guidelines outlined in the Preferred Reporting Items for Systematic Reviews and Meta-Analyses (PRISMA) statement [12]. The PRISMA checklist is provided in Table S2.

## Eligibility criteria

The inclusion criteria for this study were as follows: (1) all study designs that reported data on anemia prevalence, risk factors, or neonatal and maternal outcomes, specifically in pregnant women; (2) anemia defined as a blood hemoglobin (Hb) concentration below 11 g/dl or a hematocrit level less than 33% per the WHO [4]; (3) only studies conducted in Egypt were considered; and (4) studies published between January 1, 2010, and August 18, 2024, were included. The study period from January 1, 2010, to August 18, 2024, was selected to capture recent data and reflect the current prevalence of anemia in Egypt, along with associated risk factors and outcomes. This ensures the inclusion of up-to-date research for a comprehensive analysis.

The exclusion criteria were: (1) studies conducted on non-Egyptians; (2) preprints; and (3) studies reporting irrelevant outcomes. Two independent authors (H.K. and A.K.A.) selected relevant articles based on the specified inclusion and exclusion criteria, and the selection was cross-checked by A.B., S.I. and N.B. for accuracy and consistency.

## Definitions

In this study, we adopted the WHO definition of anemia in pregnant women, which is diagnosed when Hb levels are below 11 g/dL [4]. The WHO further categorizes anemia severity into three levels: Mild anemia, with Hb levels between 10 and 10.9 g/dL; Moderate anemia, with Hb levels between 7 and 9.9 g/dL; and Severe anemia, with Hb levels below 7 g/dL [13].

## Data extraction

Data extraction was carried out independently by three reviewers using a standardized data extraction Excel sheet (M.S.E., N.B. and M.K.), which was then cross-checked by two additional reviewers (E.A. and H.W.A.H). The extracted data included the following: last name of the first author, publication year, study period, type of study, governorate, residence (rural vs. urban), total sample size, number of pregnant women with anemia, trimester, risk factors and complications of both newborns and mothers.

## Quality assessment

We utilized the Joanna Briggs Institute (JBI) quality assessment tool to evaluate studies on anemia in pregnant women [14]. This tool includes nine key questions to assess whether the sample frame and methods were appropriate, the sample size sufficient, and the study subjects and settings adequately described. Additionally, it evaluates the validity of methods used to diagnose anemia, the reliability of measurements across participants, the appropriateness of statistical analysis, and the adequacy of the response rate, including how a low response rate was managed. Two reviewers independently (A.K.A. and A.B.) evaluated the included studies based on the aforementioned tool, and any discrepancies were resolved by M.S.E. and H.W.A.H. The checklist items for Joanna Briggs’s critical appraisal tool is presented in Table S3.

### Statistical analysis

A pooled prevalence, odds ratio (OR), and standardized mean difference (SMD), each with a 95% confidence interval (CI), were calculated using a random effects framework employing the inverse variance method. SMD values of 0.2, 0.5, and 0.8 represent small, medium, and large effects, respectively [15]. Sub-group analyses were conducted based on trimester, anemia severity and region. Heterogeneity between the studies was assessed using the I-squared (%) and Cochrane’s Q statistics [16]. The degree of heterogeneity was classified as follows: low (I² = 0–25%), moderate (I² = 26–50%), substantial (I² = 51–75%), and considerable (I² > 75%). Sensitivity analysis was performed using the leave-one-out technique to examine the robustness of the results. Publication bias was assessed through a funnel plot and Egger’s regression test. All statistical analyses were performed using R software (version 4.4.1). A p-value below 0.05 was considered indicative of statistical significance. For the assessment of publication bias, a p-value of less than 0.1 from Egger’s test was regarded as evidence of potential publication bias [17, 18].

## Results

### Characteristics of eligible articles

**Fig. 1:**
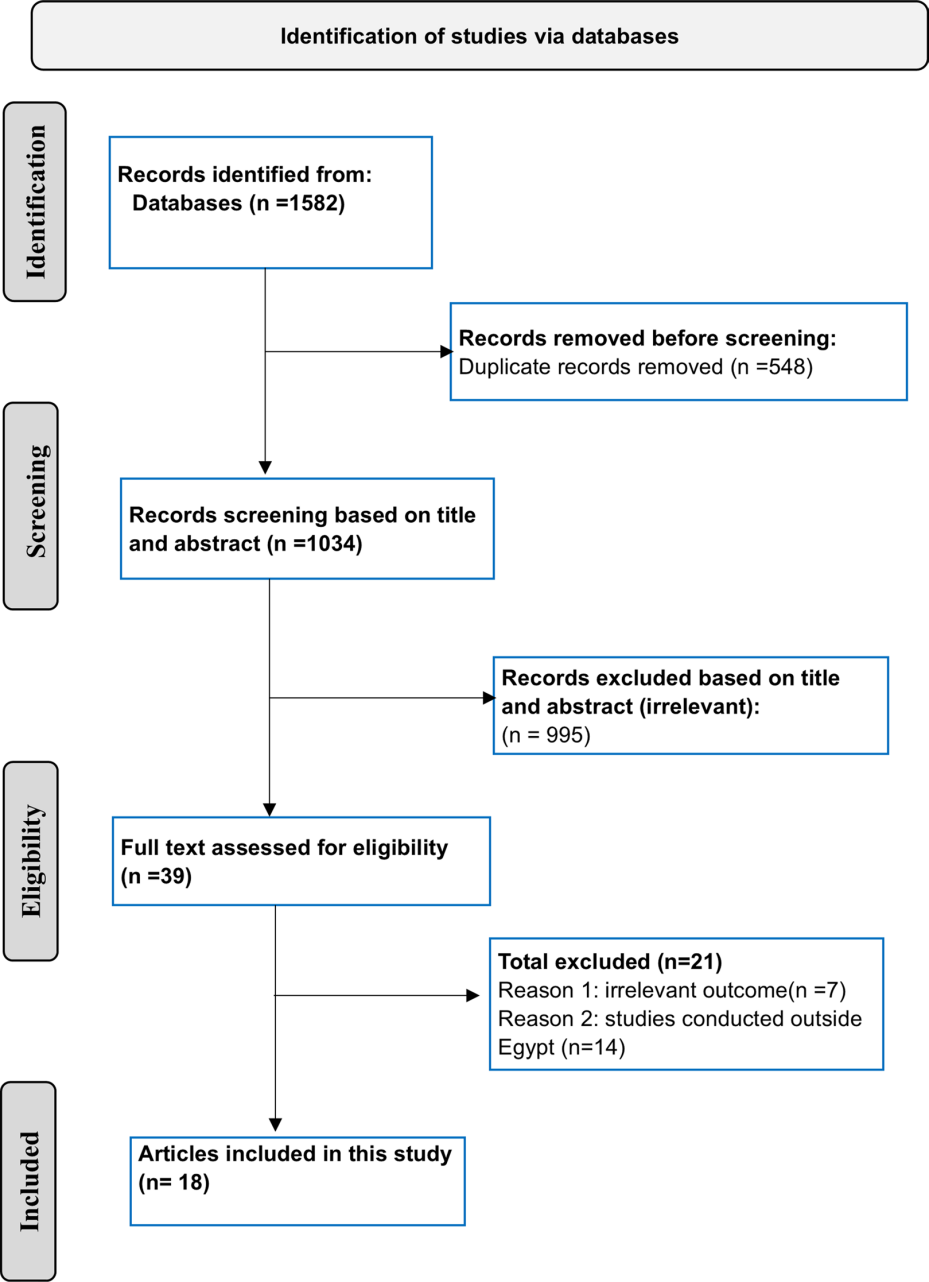
PRISMA flow diagram illustrating the selection process of the included studies

A total of 18 studies met the eligibility criteria and were included in this meta-analysis with a total sample size of 14,548, as shown in Fig. 1 [19–36]. Sixteen studies reported prevalence data on anemia among Egyptian pregnant women, while two focused on neonatal and maternal complications of anemia [22, 27]. Of these sixteen studies, ten were conducted in Lower Egypt (The northern part of Egypt) [23, 25, 26, 28, 30, 32–36], while the remaining six were from Upper Egypt (The southern part of Egypt) [19–21, 24, 29, 31]. The largest sample size was from a study in Minia Governorate, with 5,500 participants [24], while the smallest sample size was from a study in Menoufia Governorate, with 177 participants [33]. The duration of the included studies spanned from August 2010 to May 2023. Five of the included studies involved pregnant patients in the 3rd trimester [21, 23, 31, 34, 36]. Three studies involved pregnant women in the 2nd trimester [25, 29, 33]. Three studies included women in both the 2nd and 3rd trimester [19, 20, 35]. One study included pregnant women in all trimester [24]. The remaining four studies collected data without specifying the trimester [26, 28, 30, 32]. The characteristics of the included studies are presented in Table 1. The quality of the included studies is considered fair to good, with all scoring 7 or more out of 9, as shown in Table S4.

**Table 1:** The characteristics of the included studies

Last Name of the First Author (Publication Year)	Period of the Study	Type of Study	Governorate	Total Sample Size	Residence (Rural vs. Urban)	Trimester	Prevalence of anemia (%)
(a) studies reporting prevalence data on anemia among Egyptian pregnant women							
Rezk (2015) [25]	2013	CS	Menoufia	2470	Rural	2nd	51.3
Ahamed (2018) [20]	2015–2016	CS	Assiut	400	Mixed	2nd and 3rd	32.5
El-Moselhy (2017) [35]	2014–2015	CS	Kafr Al-Sheikh	200	Urban	2nd and 3rd	32.0
Abd-Elfatah (2023) [19]	2022–2023	CS	Assiut	350	Mixed	2nd and 3rd	30.9
Ibrahim (2022) [23]	2019–2020	PC	Menoufia	300	Mixed	3rd	66.7
El-Shazly (2016) [33]	2013–2014	CS	Menoufia	177	Mixed	2nd	52.5
Elzeiny (2019) [36]	2017–2017	CS and PC	Alexandria	206	Urban	3rd	73.8
Mostafa (2022) [24]	2019–2021	CS and PC	Minia	5500	Mixed	1st, 2nd, and 3rd	40.2
Ali (2023)[30]	2018–2018	CS	Sharqia	269	Mixed	N/A	39.0
El-Ashiry (2014)[31]	N/A	CS	Fayoum	381	Not specified	3rd	67.5
Eweis (2021)[21]	2019	CS	Beni-Suef	383	Mixed	3rd	72.1
Ahmed (2023)[29]	N/A	CS	Qena	1000	Mixed	2nd	37.4
El-Mahallawi (2019)[34]	2018	RC	Cairo	1552	N/A	3rd	40.9
El Sayed (2023)[32]	2019–2020	CS	Zagazig	354	N/A	N/A	43.8
Afifi (2013)[28]	2010–2011	PC	Cairo	206	N/A	N/A	46.1
Tarek (2023)[26]	2019–2019	PC	Cairo	200	Mixed	N/A	59.0
(b) studies focusing on neonatal and maternal complications of anemia in pregnant women without prevalence data							
Gomaa (2021)[22]	N/A	CS	Cairo	500	Mixed	N/A	N/A
Labib (2021)[27]	2020–2020	PC	Giza	100	Mixed	N/A	N/A

Abbreviation: N/A: Not Available, CS: Cross-Sectional, PC: Prospective Cohort, RC: Retrospective Cohort

### The overall prevalence of anemia among pregnant women in Egypt

**Fig. 2:**
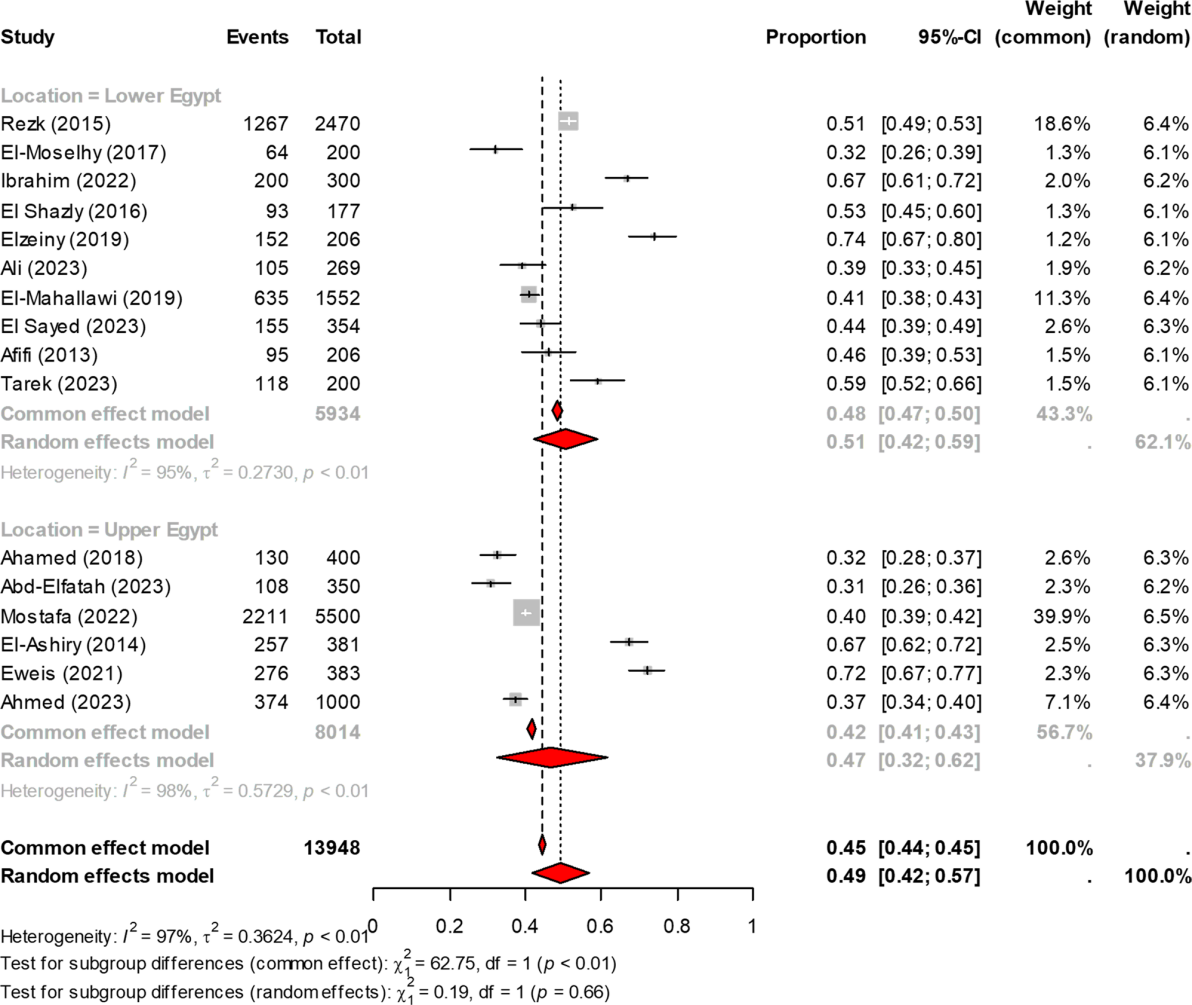
Overall prevalence of anemia among pregnant women in Egypt, stratified by region (Lower versus Upper Egypt)

**Fig. 3:**
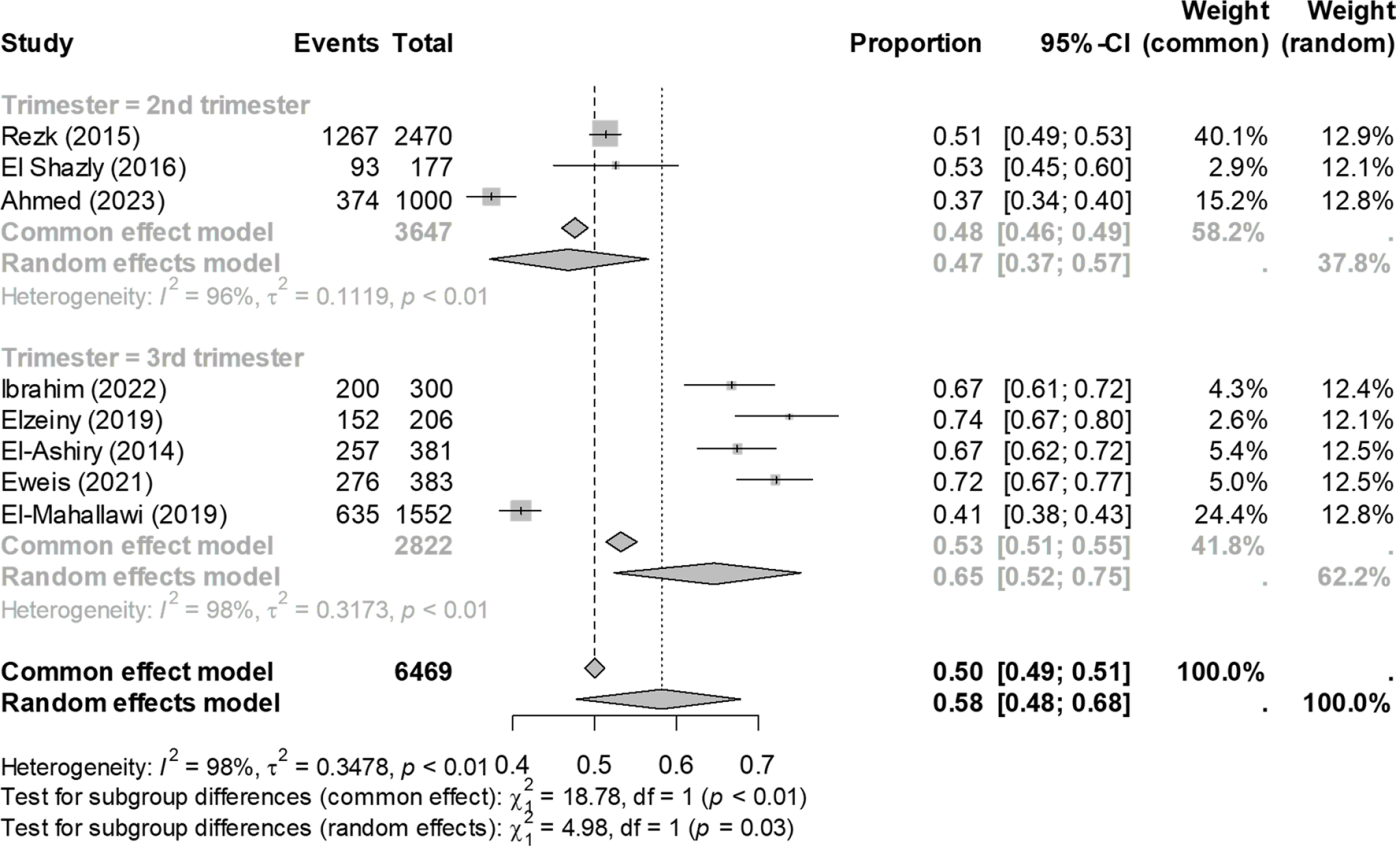
Prevalence of anemia among pregnant women in Egypt, stratified by trimester

Sixteen studies investigated the prevalence of anemia among pregnant women in Egypt, with a total sample size of 13,948. The Overall prevalence of anemia among pregnant women in Egypt was 49% (95% CI: 42–57), as shown in Fig. 2. When stratified by region, the prevalence in Lower Egypt was estimated at 51% (95% CI: 42–59), while in Upper Egypt, the prevalence was slightly lower at 47% (95% CI: 32–62) with no statistically significant difference (*P* = 0.66). When analyzed by trimester, anemia in the second trimester showed a lower pooled prevalence of 47% (95% CI: 37–57) compared to 65% (95% CI: 52–75) in the third trimester, with a statistically significant difference (*P* = 0.03), as depicted in Fig. 3. Among pregnant women diagnosed with anemia, stratification by severity revealed that the majority of cases were mild and moderate anemia, with few cases of severe anemia. The prevalence was 47% for mild anemia (95% CI: 33–61), 47% for moderate anemia (95% CI: 37–58), and 3% for severe anemia (95% CI: 1–11), as shown in Fig. S1. These differences were statistically significant (*p* < 0.001). Overall, the heterogeneity was considerable, as evidenced by an I² statistic exceeding 75%.

### Determinants of anemia among pregnant women in Egypt

**Fig. 4:**
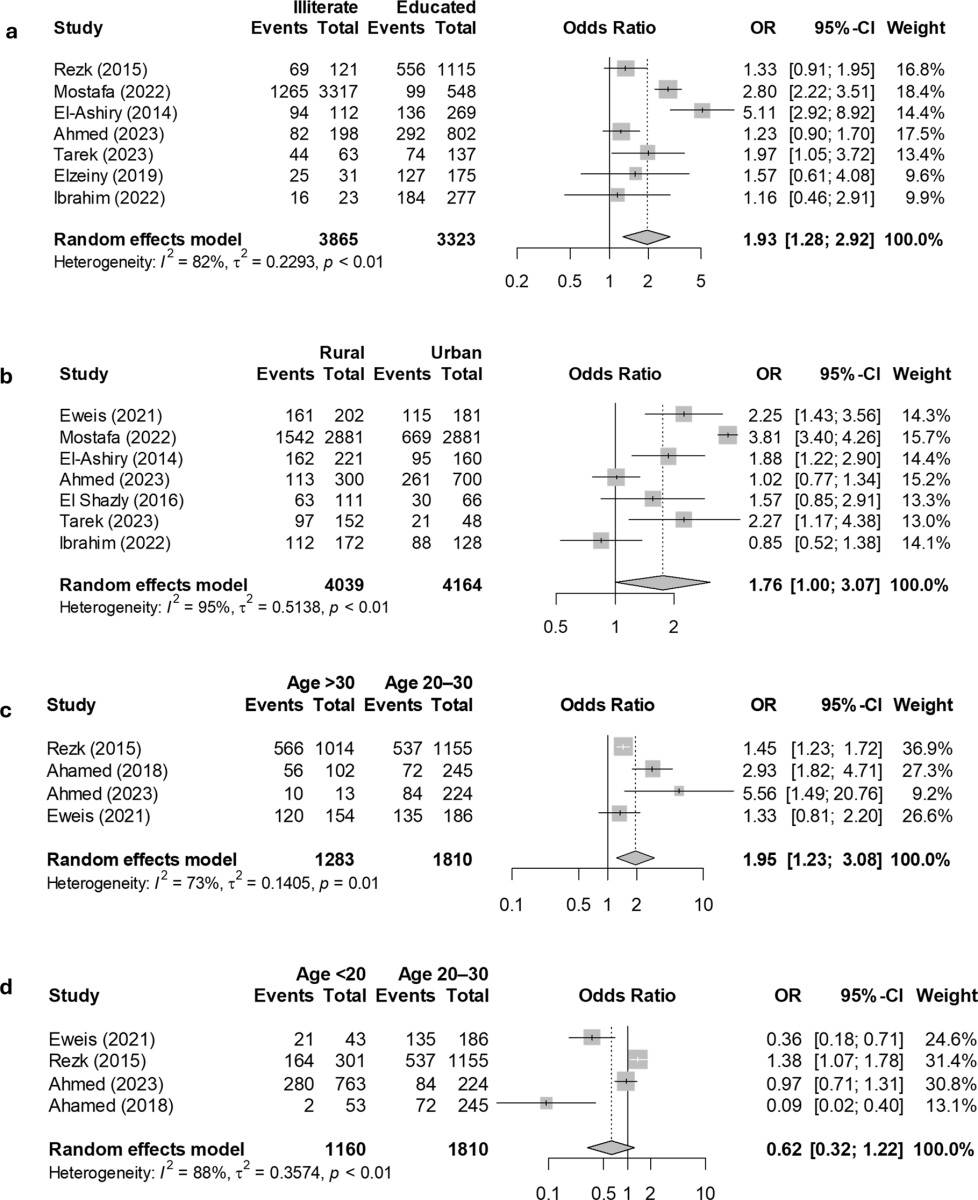
Determinants of Anemia Among Pregnant Women in Egypt: Forest plots display a meta-analysis of odds ratios for the following comparisons: (**a**) illiterate versus educated, (**b**) rural versus urban residence, (**c**) women over 30 years versus those aged 20–30, and (**d**) women under 20 years versus those aged 20–30

**Fig. 5:**
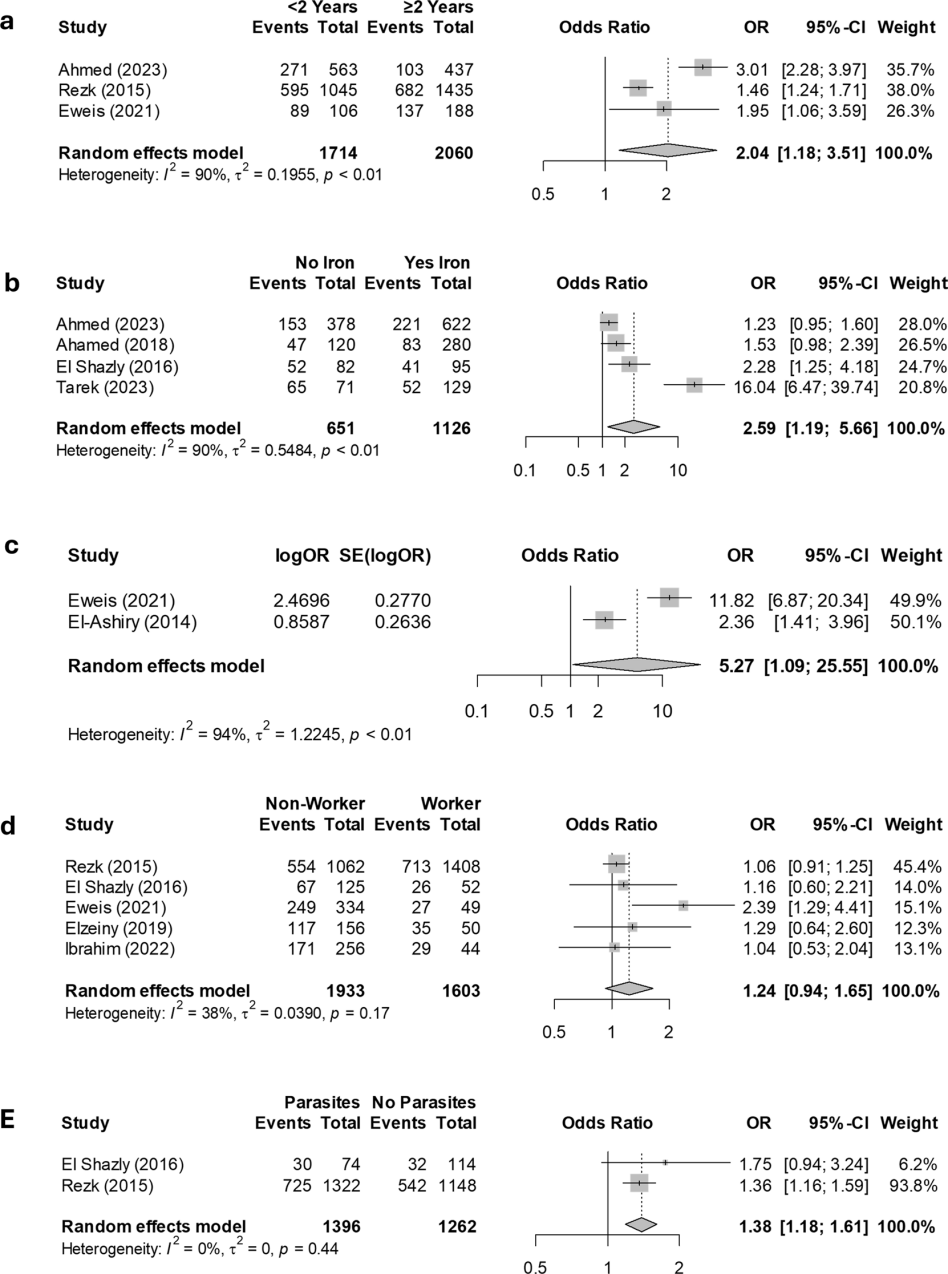
Determinants of Anemia Among Pregnant Women in Egypt: Forest plots present a meta-analysis of odds ratios for the following comparisons: (**a**) birth spacing of less than 2 years versus 2 years or more, (**b**) no iron supplementation versus iron supplementation, (**c**) antenatal care visits fewer than 5 versus 5 or more, (**d**) not working versus working, and (**e**) presence of intestinal parasites versus no intestinal parasites

Table 2 displays the meta-analysis statistics alongside the heterogeneity assessments for various determinants of anemia among pregnant women in Egypt. Women over 30 years had 1.95 times higher odds of developing anemia compared to those aged 20–30 years (OR: 1.95, 95% CI: 1.23–3.08, *P* = 0.004). Conversely, women under 20 years had lower, non-significant odds of anemia compared to the 20–30 age group (OR: 0.62, 95% CI: 0.32–1.22, *P* = 0.17). Women living in rural areas had 1.76 times higher odds of developing anemia compared to those in urban areas (OR: 1.76, 95% CI: 1.01–3.07, *P* = 0.04). Similarly, illiterate women had 1.93 times higher odds of developing anemia than their educated counterparts (OR: 1.93, 95% CI: 1.28–2.92, *P* = 0.002). The odds of developing anemia were not significantly different between women who were not working and those who were (OR: 1.24, 95% CI: 0.94–1.65, *P* = 0.13). However, women with a birth spacing of two years or less had 2.04 times higher odds of developing anemia compared to those with birth spacing longer than two years (OR: 2.04, 95% CI: 1.18–3.51, *P* = 0.01). Women who did not take iron supplementation had 2.59 times higher odds of developing anemia compared to those who took iron supplements (OR: 2.59, 95% CI: 1.19–5.66, *P* = 0.01). Additionally, pregnant women with intestinal parasites had 1.38 times higher odds of anemia compared to those without parasites (OR: 1.38, 95% CI: 1.18–1.61, *P* < 0.001). Lastly, women with fewer than five antenatal care visits had 5.27 times higher odds of anemia compared to those with five or more visits (OR: 5.27, 95% CI: 1.09–25.55, *P* = 0.043). The forest plots illustrating the meta-analysis of odds ratios for the determinants of anemia in Egypt are presented in Figs. 4 and 5. In addition, low family income [21, 23, 24, 31] and multiparity [20, 21, 24, 25, 29, 31, 33] were consistently reported as significant factors for anemia among Egyptian women. These two factors were not included in the meta-analysis due to inconsistent reference groups used across the studies, which prevented the pooling of data for meta-analysis.

**Table 2:** Meta-analysis statistics of determinants of anemia among pregnant women in Egypt

Risk factors	Included studies	Pooled odds ratio, 95%Ci, *P*-value	Heterogeneity testing I²%, *P*-value of Cochrane Q
(a) Significant factors			
Age > 30 Vs. age 20–30	4 [20, 21, 25, 29]	1.95 (1.23–3.08), 0.004	73, 0.01
Rural vs. Urban	7 [21, 23, 24, 26, 29, 31, 33]	1.76 (1.01–3.07), 0.04	95, < 0.01
Illiterate vs. Educated	7 [23–26, 29, 31, 36]	1.93 (1.28–2.92), 0.002	82, < 0.01
Brith spacing ≤ 2 years vs. > 2 years	3 [21, 25, 29]	2.04 (1.18 − 3.51), 0.01	90, < 0.01
No iron supplementation vs. taking iron supplementation	4[20, 26, 32, 33]	2.59(1.19–5.66), 0.01	90, < 0.01
Antenatal care visits < 5 vs. ≥ 5	2[21, 31]	5.27(1.09–25.55), 0.043	94, < 0.01
Intestinal parasites vs. No Intestinal parasites	2[25, 33]	1.38 (1.18–1.61), < 0.001	0, 0.44
(b) Insignificant factors			
Age < 20 Vs. age 20–30	4 [20, 21, 25, 29]	0.62 (0.32 − 1.22), 0.17	88, < 0.01
Not Working vs. Working	5[21, 23, 25, 33, 36]	1.24 (0.94–1.65), 0.13	38, 0.17

### Dietary factors associated with anemia among pregnant women in Egypt

The specific study findings and the association of each dietary factor with anemia among pregnant women in Egypt are detailed in Table 3. Overall, Low intake of meat, vegetables, fruits, fish, and chicken was consistently associated with a higher risk of anemia across studies, while high tea intake was linked to an increased risk of anemia. In addition, limited consumption of milk and liver was identified as significant dietary factors. Milk intake less than 2 times per week was significantly associated with anemia, as reported in one study (*P* < 0.001) [24]. Similarly, consuming liver less than two servings per week was significantly associated with anemia (*P* = 0.021) [33].

**Table 3:** Dietary factors associated with anemia among pregnant women in Egypt

Dietary Factors	Specific Study Findings
Meat intake (4 studies: [21, 24, 31, 33])	Consuming meat fewer than two times per week was significantly associated with anemia (*P* < 0.001) [24].
	Eating meat less than once per week increased the odds of anemia (OR: 2.13, 95% CI: 1.2–3.7, *P* = 0.008) [31].
	Consuming red meat less than two servings per week was strongly linked to anemia (*P* = 0.001), while white meat intake below this threshold also showed a significant association (*P* < 0.001) [33].
	Eating meat less than once per month was strongly associated with anemia (OR: 52.1, *P* < 0.001) [21].
Vegetable and Fruit intake (5 studies: [21, 24, 25, 31, 33])	Consuming vegetables fewer than two times per week was significantly linked to anemia (*P* < 0.001) [24].
	Eating fruits less than four times per week increased anemia risk (OR: 1.9, 95% CI: 1.1–3.4, *P* = 0.02) [31].
	Green vegetable intake below two servings per week was significantly associated with anemia (*P* < 0.001), as was fresh fruit consumption below this level (*P* < 0.001) [33].
	Combined vegetable and fruit intake less than three times per week was linked to anemia (*P* < 0.01) [25].
	Eating vegetables fewer than four servings per week was strongly associated with anemia (OR: 14.966, *P* < 0.001), while fruit intake fewer than four servings per week showed an even higher association (OR: 15.632, *P* < 0.001) [21]
Fish and Chicken intake (1 study: [21])	Consuming fish less than once per week was associated with an increased risk of anemia (OR: 7.9, *P* = 0.035). Similarly, eating chicken less than once per week showed a strong association with anemia (OR: 16.173, *P* < 0.001) [21].
Egg intake (2 studies: [24, 33])	Study [24] found that consuming eggs fewer than two times per week was significantly linked to anemia (*P* < 0.001). However, Study [33] reported that consuming fewer than two servings of eggs per week was not significantly associated with anemia (*P* = 0.468).
Tea intake (3 studies: [21, 31, 33])	Drinking tea more than once per day was strongly associated with anemia (OR: 113.9, *P* < 0.001) [21, 31, 33].
Milk intake (1 study: [24])	Milk consumption less than 2 times per week was significantly associated with anemia (*P* < 0.001) [24].
Liver consumption (1 study: [33])	Eating liver less than two servings per week was significantly associated with anemia (*P* = 0.021)[33].

### Neonatal complications in infants born to anemic pregnant women in Egypt

**Fig. 6:**
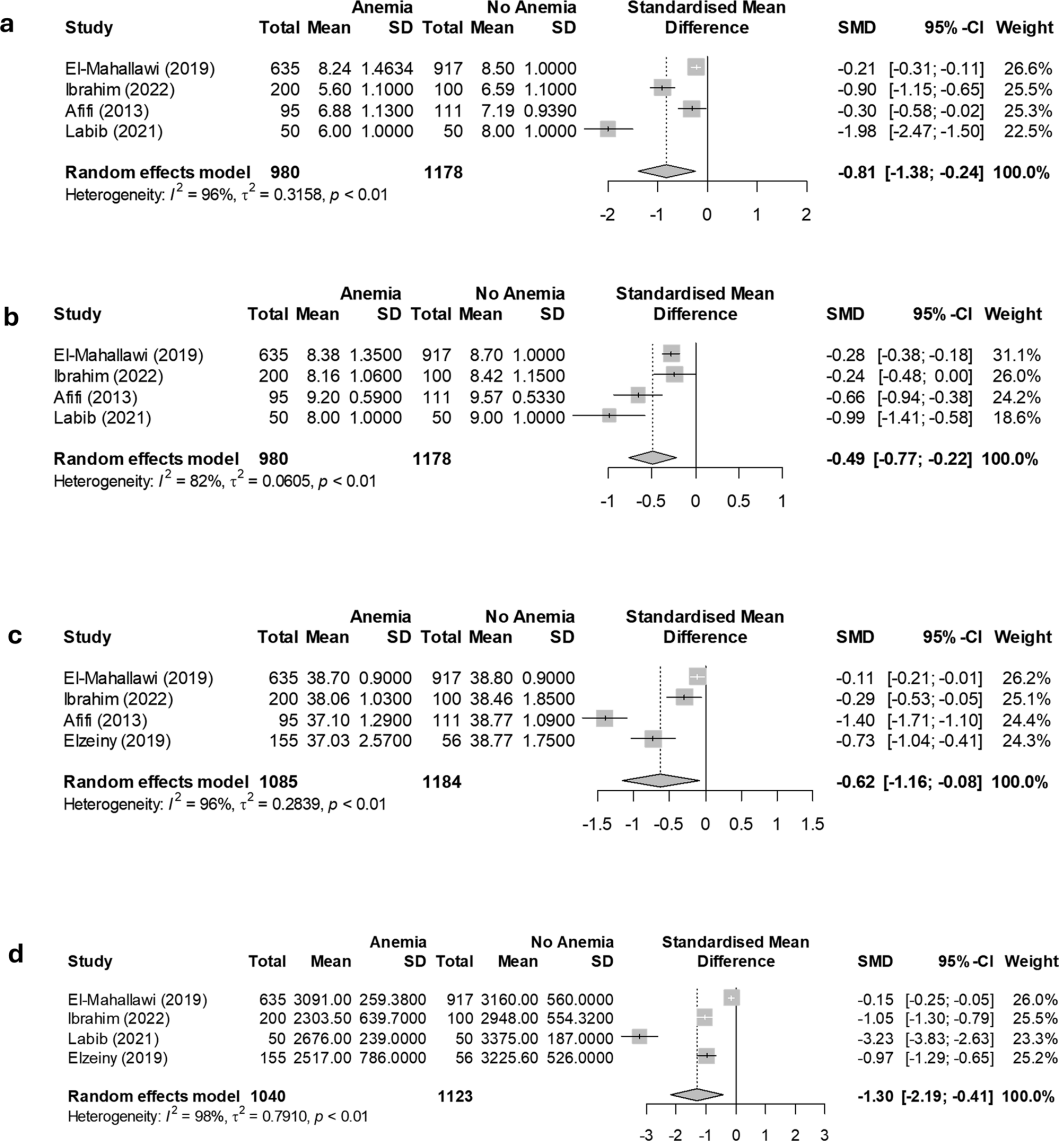
Analysis of standardized mean differences in neonatal complications between anemic and non-anemic pregnant women, including (**a**) Apgar score at 1 min, (**b**) Apgar score at 5 min, (**c**) Gestational age, and (**d**) Weight at delivery

The meta-analysis statistics of neonatal complications related to anemia in pregnant women are detailed in Table 4. Pregnant women with anemia exhibited significantly lower Apgar scores at both 1 min and 5 min, with SMDs of -0.81 (95% CI: -1.38 to -0.24, *P* = 0.006) and − 0.49 (95% CI: -0.77 to -0.22, *P* < 0.001), respectively. Gestational age and weight at delivery also showed significant reductions, with SMDs of -0.62 (95% CI: -1.16 to -0.08, *P* = 0.023) and − 1.3 (95% CI: -2.19 to -0.41, *P* = 0.004), respectively, indicating that weight at delivery was most negatively impacted. Figure 6 displays the meta-analysis of the SMDs in neonatal complications between anemic pregnant women and non-anemic women. Additionally, studies identified a higher incidence of SGA infants [21, 23], and one study found that 73.5% of severe anemia cases resulted in LBW [22]. Regarding NICU admissions, one study reported a higher but non-significant incidence (*p* = 0.14) [23], while others found significantly higher admission rates (*p* < 0.05) [34, 36]. Smaller head circumferences were observed in neonates of anemic mothers (*p* < 0.01) [36], and one study found a significantly higher neonatal death rate in cases of severe anemia (*p* < 0.001) [34].

**Table 4:** Meta-analysis of neonatal complications associated with anemia among pregnant women in Egypt

Neonatal Complications	Number of Included studies	Standardized mean difference (95% CI), *P*-value	Heterogeneity testing I²%, *P*-value of Cochrane Q
Apgar at 1 min	4, [23, 27, 28, 34]	-0.81(-1.38 to -0.24), 0.006	96, < 0.01
Apgar at 5 min	4, [21, 29, 33, 34]	-0.49(-0.77 to -0.22), < 0.001	82, < 0.01
Gestational age	4, [23, 28, 34, 36]	-0.62(-1.16 to -0.08), 0.023	96, < 0.01
Weight at delivery	4, [23, 27, 34, 36]	-1.3(-2.19 to -0.41), 0.004	98, < 0.01

### Maternal complications among anemic pregnant women in Egypt

Several maternal complications were reported among anemic pregnant women in Egypt. Immediate postpartum hemorrhage was observed at significantly higher rates in two studies(*p* < 0.05) [23, 34]. One study noted a significantly higher amount of blood loss during hemorrhage in anemic mothers and a higher incidence of infections among anemic pregnant women (*p* < 0.05) [36]. Furthermore, a significantly higher frequency of ICU admissions (*p* < 0.001) and the universal requirement for blood transfusions in cases of anemia were documented [34].

### Sensitivity analysis using leave one out approach and publication bias testing

**Fig. 7:**
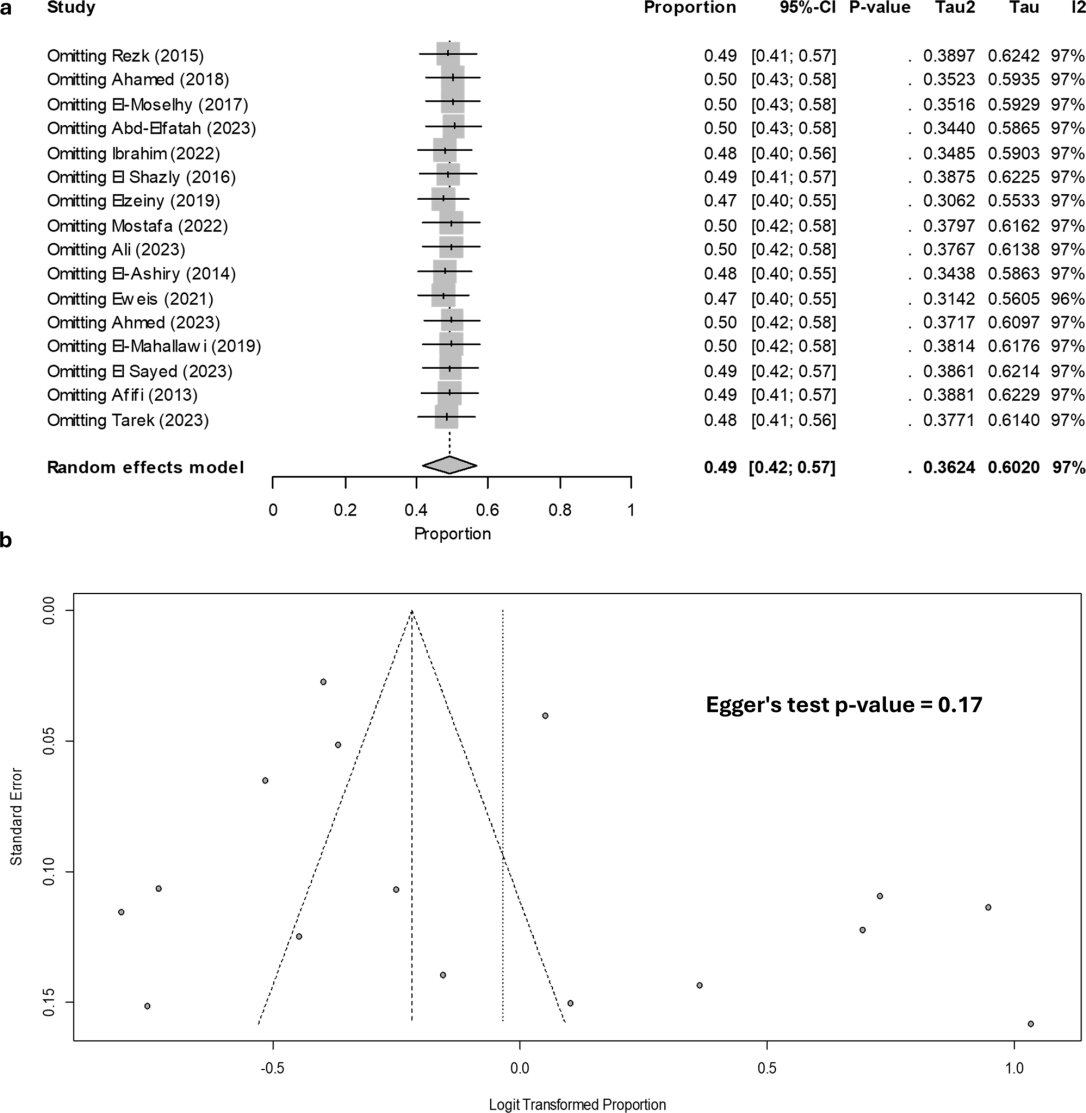
Sensitivity analysis and publication bias assessment. (**a**) Sensitivity analysis using the leave-one-out approach and (**b**) publication bias assessment through funnel plot and Egger’s regression test

Our sensitivity analysis demonstrated that the pooled estimate was both reliable and robust. No significant outliers were identified. None of the studies exhibited a change in pooled estimate greater than 2%. Additionally, there is no evidence of publication bias, as indicated by Egger’s regression test, with a p-value of 0.17. These analyses are illustrated in Fig. 7.

## Discussion

This meta-analysis is the first comprehensive examination of anemia among pregnant women in Egypt, revealing a high prevalence of 49% across the country, with no significant difference between Upper and Lower Egypt. Key determinants of anemia included maternal age over 30, rural residence, illiteracy, short birth spacing, lack of iron supplementation, presence of intestinal parasites, limited antenatal care, multiparity, and low income. Dietary factors also played a role, with a low intake of meat, vegetables, fruits, and high tea consumption consistently linked to a higher risk of anemia. Regarding neonatal complications, infants born to anemic mothers had significantly lower Apgar scores, gestational ages, and birth weights. These findings highlight the need for targeted interventions to address these determinants, aiming to reduce anemia prevalence and mitigate its adverse outcomes.

Our findings revealed an overall prevalence of 49% (95% CI: 42–57), which, according to WHO classification, represents a significant public health concern [4]. Compared to country-level meta-analyses, the prevalence in Egypt is slightly lower than in Sudan (53.0%) [10] but higher than the rates reported in Ethiopia (31.6%) [9], South Africa (31%) [37], and Iran (15%) [11]. These regional disparities may stem from differences in knowledge and attitudes toward prenatal care and anemia prevention, as well as variations in socioeconomic status and the presence of endemic diseases, all of which contribute to fluctuations in anemia prevalence.

This meta-analysis revealed increased odds of anemia among women over 30 (OR = 1.95, *p* = 0.004), consistent with previous studies [38–40]. The higher prevalence may be attributed to factors such as multiple pregnancies, age-related complications, and an increased risk of comorbidities that can predispose them to anemia. Similarly, pregnant women residing in rural areas (OR = 1.76, *p* = 0.04), or those who are illiterate(OR = 1.93, *p* = 0.002) had higher odds of anemia, consistent with findings from studies [39–42]. Rural residents often face obstacles such as limited access to healthcare and socioeconomic challenges. Additionally, illiterate women may lack the health awareness needed to recognize and address nutritional deficiencies. Factors such as short birth spacing (OR = 2.04, *p* = 0.01), lack of iron supplementation (OR = 2.59, *p* = 0.01), intestinal parasites (OR = 1.38, *p* < 0.001), and fewer antenatal visits (OR = 5.27, *p* = 0.043) were also associated with higher odds of anemia. A systematic review examining the impact of birth spacing on maternal and child nutritional status indicated that shorter birth intervals were associated with an increased risk of maternal anemia [43]. Intestinal parasites were also strongly linked to maternal anemia, as reported in this study, which aligns with previous research [44–47]. Parasitic infections can significantly impact hemoglobin levels and lead to anemia by hindering iron absorption in the intestines and depleting red blood cells.

In this study, newborns of anemic pregnant women had significantly lower Apgar scores at both 1 and 5 min, with SMDs of -0.81 (*P* = 0.006) and − 0.49 (*P* < 0.001), respectively. These findings align with other research showing notably lower Apgar scores in newborns of anemic mothers compared to those of non-anemic mothers [7, 48]. Apgar score is a standard measure used to assess a baby’s health shortly after birth. A lower Apgar score indicates potential issues with the newborn’s heart rate, breathing, muscle tone, reflex response, or color, suggesting that maternal anemia may negatively affect newborn health outcomes. A potential mechanism is that maternal anemia reduces oxygen supply to the fetus, leading to fetal hypoxia, which can trigger fetal anaerobic metabolism, causing lactic acid buildup that overwhelms the fetal buffering system and results in metabolic acidosis [49]. This acidosis disrupts essential functions like heart rate and breathing, leading to lower Apgar scores [49]. Additionally, maternal anemia can impair fetal growth, increasing the risk of low birth weight and preterm birth, both of which are linked to lower Apgar scores [50].

Furthermore, neonates born to anemic mothers had lower gestational age and birth weight compared to those born to non-anemic mothers, with SMDs of -0.62 (*p* = 0.023) and − 1.3 (*p* = 0.004), respectively. This indicates that birth weight was the most adversely affected among the tested outcomes. A recent meta-analysis examined the effects of maternal anemia on neonatal birth weight, demonstrating that anemic pregnant women have a significantly elevated risk of delivering LBW neonates compared to non-anemic mothers [51]. Similarly, multiple studies have highlighted the association between maternal anemia and a higher incidence of neonatal prematurity [48, 52–54]. In this study, weight at delivery demonstrated an SMD of -1.3 (*P* = 0.004), highlighting it as the neonatal parameter most significantly impacted by maternal anemia. Maternal anemia results in reduced red blood cell count and hemoglobin levels, both critical for oxygen transport, which can impair fetal growth more directly and substantially than other neonatal parameters. Additionally, several maternal complications, including significantly higher rates of immediate postpartum hemorrhage, increased infection incidence, and more frequent ICU admissions, have been reported [23, 34, 36]. Previous studies confirm these findings, showing that anemic pregnant women have higher ICU admission rates, more frequent infections, increased postpartum hemorrhage, and a greater need for blood transfusions [6, 55–59].

### Implications for policy and practice

This meta-analysis highlights the high prevalence of anemia among pregnant women in Egypt, and its adverse effects on neonatal health. Addressing this issue requires policy measures that prioritize expanding access to iron supplementation and antenatal care, especially in rural areas. Educational initiatives are crucial to enhance maternal health literacy on nutrition and birth spacing, while public health campaigns targeting parasite control can help reduce anemia risk. Given the impacts on infant health, enhanced neonatal support is essential for babies born to anemic mothers. Additionally, targeted interventions for women over 30 and strengthened family planning support can further decrease anemia rates, improving maternal and infant health outcomes.

### Strengths and limitations

A key strength of this analysis is its rigorous methodology, providing a thorough evaluation of anemia prevalence, determinants, and associated complications among pregnant women in Egypt. It incorporates recent data to ensure that findings reflect contemporary prevalence and risk factors. Additionally, the fair quality of the studies strengthens the reliability of this analysis. Sensitivity analysis confirms the absence of significant outliers, and the lack of publication bias further underscores the analysis’s robustness. However, it is important to acknowledge certain limitations within this study. Primarily, the lack of prevalence data from specific regions in Egypt limits the scope and potentially the applicability of our conclusions. Furthermore, the scarcity of studies assessing neonatal and maternal outcomes hampers comprehensive meta-analyses. Another limitation is that this study was not pre-registered. However, we ensured methodological rigor by strictly adhering to the PRISMA guidelines. Finally, high heterogeneity was observed in this meta-analysis, which is inherent and expected in meta-analyses of prevalence data [60, 61]. This variability may be partially explained by differences in cohort characteristics, such as maternal age, place of residence, literacy levels, birth spacing, iron supplementation status, antenatal care access, parity, and socioeconomic conditions. These factors are likely key contributors to the observed heterogeneity. These limitations underscore the necessity for additional research to comprehensively address these knowledge gaps.

## Conclusion

Our findings reveal a 49% prevalence of anemia among pregnant women in Egypt, indicating a serious public health concern based on WHO criteria. Key determinants include maternal age over 30, rural residence, illiteracy, short birth spacing, insufficient iron supplementation, intestinal parasites, multiparity, low income, and limited antenatal care. Additionally, infants born to anemic mothers showed significantly lower Apgar scores, gestational ages, and birth weights. These findings underscore the urgent need for targeted interventions to address these determinants and reduce the prevalence of anemia.

## Electronic supplementary material

Below is the link to the electronic supplementary material.

Supplementary Material 1

## Data Availability

All data generated and analyzed throughout this study were included either in this article or its supplementary information file.

## Abbreviations

WHO: World Health Organization
LBW: Low Birth Weight
SGA: Small for Gestational Age
Hb: Hemoglobin
PRISMA: Preferred Reporting Items for Systematic Reviews and Meta-Analyses
JBI: Joanna Briggs Institute
OR: Odds Ratio
SMD: Standardized Mean Difference
CI: Confidence Interval
ICU: Intensive Care Unit
NICU: Neonatal Intensive Care Unit
