# Supplementary material for: Anemia in pregnancy: a systematic review and meta-analysis of prevalence, determinants, and health impacts in Egypt
Source: BMC Pregnancy Childbirth. 2025 Jan 14;25:29. doi: 10.1186/s12884-024-07111-9 (PMC11731563; doi:10.1186/s12884-024-07111-9)
Supplement: Supplementary file 1 — Supplementary Material 1 [file 12884_2024_7111_MOESM1_ESM.docx]

**Supplementary file**

**Anemia in Pregnancy: A Systematic Review and Meta-Analysis of Prevalence, Determinants, and Health Impacts in Egypt**

Ahmed Azzam^1^*, Heba Khaled^2^, Alrefaey K. Alrefaey^3^, Amar Basil^4^, Sarah Ibrahim^5^, Mohamed S. Elsayed^6^, Muhammad Khattab^7^, Nashwa Nabil^8^, Esraa Abdalwanees^9^, Hala Waheed Abdel Halim^10^

^1^Department of Microbiology and Immunology, Faculty of Pharmacy, Helwan University, Cairo, Egypt

^2^Department of Biochemistry, Faculty of Pharmacy, Cairo University, Cairo, Egypt

^3^Department of Anesthesia, Intensive Care, and Pain Management, Faculty of Medicine, Mansoura University, Mansoura, Egypt

^4^Intern, Bashir Hospital, Amman, Jordan

^5^Family Medicine Center, Downtown Cairo Medical Administration, Cairo Medical Directorate, Cairo, Egypt

^6^Pharmacist, Mansoura University Children’s Hospital, Mansoura, Egypt

^7^Specialty Doctor in Surgery, Yeovil District Hospital, Somerset NHS Foundation Trust, Somerset, UK

^8^Department of Community, Environmental and Occupational Medicine, Faculty of Medicine, Benha University, Egypt

^9^Pediatric Specialist, Ain Alkhaleej Hospital, Al Ain City, Abu Dhabi, UAE

^10^Department of Obstetrics and Gynecology, Faculty of Medicine for Girls, Al-Azhar University, Cairo, Egypt

**Table S1:** Detailed search strategy for each database

| Database | Keywords |
| --- | --- |
| PubMed | (Anemia[tiab] OR Anaemia[tiab] OR Hemoglobin[tiab] OR Haemoglobin[tiab] OR "Iron Deficiency"[tiab])  AND (Pregnan*[tiab] OR Maternal[tiab] OR Prenatal[tiab] OR Gestating[tiab] OR "Pregnant Women"[tiab] OR Antenatal[tiab])  AND Egypt*[tiab]  AND ("2010"[Date - Publication] : "2024"[Date - Publication]) |
| Scopus | ((TITLE-ABS-KEY(Anemia OR Anaemia OR Hemoglobin OR Haemoglobin OR "Iron Deficiency"))  AND (TITLE-ABS-KEY(Pregnan* OR Maternal OR Prenatal OR Gestating OR "Pregnant Women" OR Antenatal))  AND (TITLE-ABS-KEY(Egypt*))  AND PUBYEAR > 2009 AND PUBYEAR < 2025 |
| Web of Science | 1: ((((TS=(Anemia )) OR TS=(Anaemia )) OR TS=(Hemoglobin )) OR TS=(Haemoglobin )) OR TS=("Iron Deficiency")  2: ((((TS=(Anemia )) OR TS=(Anaemia )) OR TS=(Hemoglobin )) OR TS=(Haemoglobin )) OR TS=("Iron Deficiency")  3: ((((((TS=(Pregnan*)) OR TS=(Maternal))) OR TS=(Prenatal)) OR TS=(Gestating)) OR TS=("Pregnant Women")) OR TS=(Antenatal)  4: TS=(Egypt*)  5: #2 AND #3 AND #4  6: #2 AND #3 AND #4 and 2024 or 2023 or 2022 or 2021 or 2020 or 2019 or 2018 or 2017 or 2016 or 2015 or 2014 or 2013 or 2012 or 2011 or 2010 (Publication Years) |
| Google Scholar, and the Egyptian Knowledge Bank | (Anemia OR Anaemia OR Hemoglobin OR Haemoglobin OR "Iron Deficiency") AND (Pregnancy* OR Maternal OR Prenatal OR Gestating OR "Pregnant Women" OR Antenatal) AND (Egypt*) |

**Table S2:** Supplementary preferred reporting items for systematic reviews and meta-analyses (PRISMA) checklist

| Section/topic | Item No | Checklist item | Reported on page No |
| --- | --- | --- | --- |
| Title | 1 | Identify the report as a systematic review, meta-analysis, or both | 1 |
| Abstract | | | |
| Structured summary | 2 | Provide a structured summary including, if applicable, background, objectives, data sources, study eligibility criteria, participants, interventions, study appraisal and synthesis methods, results, limitations, conclusions and implications of key findings, systematic review registration number | 2,3 |
| Introduction | | | |
| Rationale | 3 | Describe the rationale for the review in the context of what is already known | 4 |
| Objectives | 4 | Provide an explicit statement of questions being addressed with reference to participants, interventions, comparisons, outcomes, and study design (PICOS) | 4 |
| Methods | | | |
| Protocol and registration | 5 | Indicate if a review protocol exists, if and where it can be accessed (such as web address), and, if available, provide registration information including registration number | Not registered |
| Eligibility criteria | 6 | Specify study characteristics (such as PICOS, length of follow-up) and report characteristics (such as years considered, language, publication status) used as criteria for eligibility, giving rationale | 4, 5 |
| Information sources | 7 | Describe all information sources (such as databases with dates of coverage, contact with study authors to identify additional studies) in the search and date last searched | 4, 5 |
| Search | 8 | Present a full electronic search strategy for at least one database, including any limits used, such that it could be repeated | 4, 5 |
| Study selection | 9 | State the process for selecting studies (that is, screening, eligibility, included in the systematic review, and, if applicable, included in the meta-analysis) | 4, 5 |
| Data collection process | 10 | Describe the method of data extraction from reports (such as piloted forms, independently, in duplicate) and any processes for obtaining and confirming data from investigators | 5 |
| Data items | 11 | List and define all variables for which data were sought (such as PICOS, and funding sources) and any assumptions and simplifications made | 5 |
| Risk of bias in individual studies | 12 | Describe methods used for assessing the risk of bias in individual studies (including specification of whether this was done at the study or outcome level), and how this information is to be used in any data synthesis | 5  **Tables S3&4** |
| Summary measures | 13 | State the principal summary measures (such as risk ratio, and difference in means). | 6 |
| Synthesis of results | 14 | Describe the methods of handling data and combining results of studies, if done, including measures of consistency (such as I^2^ statistic) for each meta-analysis | 6 |
| Risk of bias across studies | 15 | Specify any assessment of risk of bias that may affect the cumulative evidence (such as publication bias, selective reporting within studies) | 6 |
| Additional analyses | 16 | Describe methods of additional analyses (such as sensitivity or subgroup analyses, meta-regression), if done, indicating which were pre-specified | 6 |
| Results | | | |
| Study selection | 17 | Give numbers of studies screened, assessed for eligibility, and included in the review, with reasons for exclusions at each stage, ideally with a flow diagram | 7  **Fig. 1** |
| Study characteristics | 18 | For each study, present characteristics for which data were extracted (such as study size, PICOS, follow-up period) and provide the citations | 7  **Table 1** |
| Risk of bias within studies | 19 | Present data on the risk of bias of each study and, if available, any outcome-level assessment (see item 12). | **Table S4** |
| Results of individual studies | 20 | For all outcomes considered (benefits or harms), present for each study (a) simple summary data for each intervention group and (b) effect estimates and confidence intervals, ideally with a forest plot | 8-14  Figs. 2-7 and Tables 2&3&4 |
| Synthesis of results | 21 | Present results of each meta-analysis done, including confidence intervals and measures of consistency | 8-14  Figs. 2-7 and Tables 2&3&4 |
| Risk of bias across studies | 22 | Present results of any assessment of risk of bias across studies (see item 15) | 14 Fig. 7 |
| Additional analysis | 23 | Give results of additional analyses, if done (such as sensitivity or subgroup analyses, meta-regression) (see item 16) | 14 Fig. 7 |
| Discussion | | | |
| Summary of evidence | 24 | Summarize the main findings including the strength of evidence for each main outcome; consider their relevance to key groups (such as health care providers, users, and policymakers) | 14 |
| Limitations | 25 | Discuss limitations at the study and outcome level (such as the risk of bias), and at the review level (such as incomplete retrieval of identified research, reporting bias) | 14&15 |
| Conclusions | 26 | Provide a general interpretation of the results in the context of other evidence, and implications for future research | 16&17 |
| Funding | | | |
| Funding | 27 | Describe sources of funding for the systematic review and other support (such as the supply of data) and the role of funders for the systematic review | 19 |

**Table S3:** The checklist items for Joanna Briggs's critical appraisal tool for prevalence studies

|  | Yes | No | Unclear | Not applicable |
| --- | --- | --- | --- | --- |
| 1. Was the sample frame appropriate to address the target population? | □ | □ | □ | □ |
| 1. Were study participants sampled in an appropriate way? | □ | □ | □ | □ |
| 1. Was the sample size adequate? | □ | □ | □ | □ |
| 1. Were the study subjects and the setting described in detail? | □ | □ | □ | □ |
| 1. Was the data analysis conducted with sufficient coverage of the identified sample? | □ | □ | □ | □ |
| 1. Were valid methods used for the identification of the condition? | □ | □ | □ | □ |
| 1. Was the condition measured in a standard, reliable way for all participants? | □ | □ | □ | □ |
| 1. Was there appropriate statistical analysis? | □ | □ | □ | □ |
| 1. Was the response rate adequate, and if not, was the low response rate managed appropriately? | □ | □ | □ | □ |

**Table S4:** Quality assessment of the included studies

| **Last Name of the First Author (Publication Year)** | **Q1** | **Q2** | **Q3** | **Q4** | **Q5** | **Q6** | **Q7** | **Q8** | **Q9** | **Out of 9** |
| --- | --- | --- | --- | --- | --- | --- | --- | --- | --- | --- |
| Rezk (2015) | 1 | 1 | 1 | 1 | 1 | 1 | 1 | 1 | 1 | 9 |
| Ahamed (2018) | 1 | 1 | 1 | 1 | 1 | 1 | 1 | 1 | 1 | 9 |
| El-Moselhy (2017) | 1 | 1 | 0 | 1 | 1 | 1 | 1 | 1 | 1 | 8 |
| Abd-Elfatah (2023) | 1 | 1 | 1 | 1 | 1 | 1 | 1 | 1 | 1 | 9 |
| Ibrahim (2022) | 1 | 1 | 0 | 1 | 1 | 1 | 1 | 1 | 1 | 8 |
| El Shazly (2016) | 1 | 1 | 0 | 1 | 1 | 1 | 1 | 1 | 1 | 8 |
| Elzeiny (2019) | 1 | 1 | 0 | 0 | 1 | 1 | 1 | 1 | 1 | 7 |
| Mostafa (2022) | 1 | 1 | 1 | 1 | 0 | 1 | 1 | 1 | 1 | 8 |
| Ali (2023) | 1 | 1 | 1 | 1 | 1 | 1 | 1 | 1 | 1 | 9 |
| El-Ashiry (2014) | 1 | 1 | 1 | 1 | 1 | 1 | 1 | 1 | 1 | 9 |
| Eweis (2021) | 1 | 1 | 1 | 1 | 1 | 1 | 1 | 1 | 1 | 9 |
| Ahmed (2023) | 1 | 1 | 0 | 1 | 1 | 1 | 1 | 1 | 1 | 8 |
| El-Mahallawi (2019) | 1 | 1 | 1 | 0 | 1 | 1 | 1 | 1 | 1 | 7 |
| El Sayed (2023) | 1 | 1 | 1 | 0 | 1 | 1 | 1 | 1 | 1 | 8 |
| Gomaa (2021) | 1 | 1 | 1 | 0 | 1 | 1 | 1 | 1 | 1 | 8 |
| Labib (2021 | 1 | 1 | 0 | 1 | 1 | 1 | 1 | 1 | 1 | 8 |
| Tarek (2023) | 1 | 1 | 0 | 1 | 1 | 1 | 1 | 1 | 1 | 8 |
| Afifi (2013) | 1 | 1 | 0 | 0 | 1 | 1 | 1 | 1 | 1 | 7 |


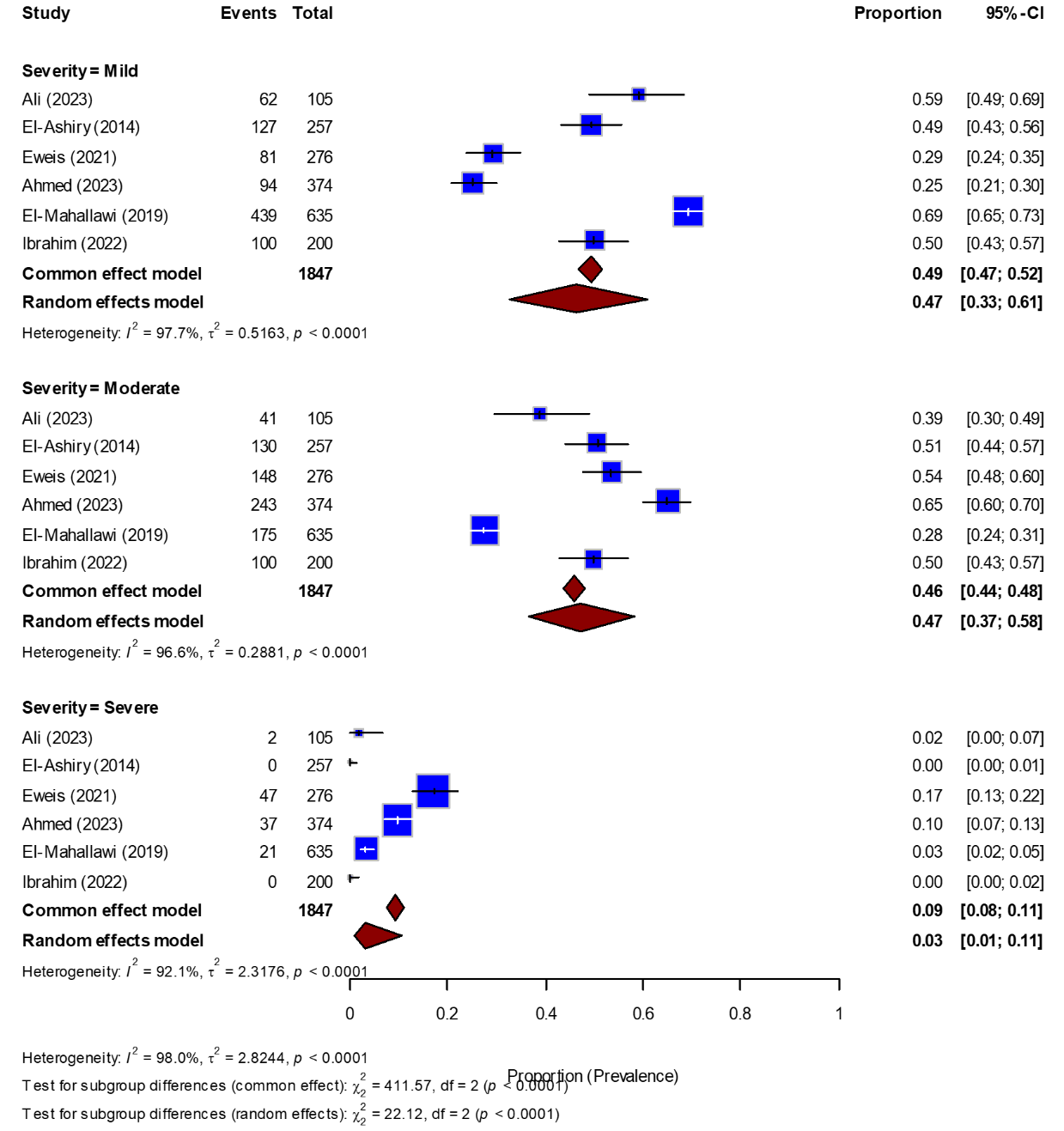


**Fig. S1: Prevalence of Anemia Among Egyptian Pregnant Women Stratified by Severity of Anemia**
